# Formation of Hydrogen Sulfide in Wine: Interactions between Copper and Sulfur Dioxide

**DOI:** 10.3390/molecules21091214

**Published:** 2016-09-10

**Authors:** Marlize Z. Bekker, Mark E. Smith, Paul A. Smith, Eric N. Wilkes

**Affiliations:** The Australian Wine Research Institute, P.O. Box 197, Glen Osmond, 5064 South Australia, Australia; mark.smith@awri.com.au (M.E.S.); paul.smith@awri.com.au (P.A.S.); eric.wilkes@awri.com.au (E.N.W.)

**Keywords:** reductive aromas, hydrogen sulfide, quinone, wine, copper, sulfur dioxide

## Abstract

The combined synergistic effects of copper (Cu^2+^) and sulfur dioxide (SO_2_) on the formation of hydrogen sulfide (H_2_S) in Verdelho and Shiraz wine samples post-bottling was studied over a 12-month period. The combined treatment of Cu^2+^ and SO_2_ significantly increased H_2_S formation in Verdelho wines samples that were not previously treated with either Cu^2+^ or SO_2_. The formation of H_2_S produced through Cu^2+^ mediated reactions was likely either: (a) directly through the interaction of SO_2_ with either Cu^2+^ or H_2_S; or (b) indirectly through the interaction of SO_2_ with other wine matrix compounds. To gain better understanding of the mechanisms responsible for the significant increases in H_2_S concentration in the Verdelho samples, the interaction between Cu^2+^ and SO_2_ was studied in a model wine matrix with and without the presence of a representative thiol quenching compound (4-methylbenzoquinone, 4MBQ). In these model studies, the importance of naturally occurring wine compounds and wine additives, such as quinones, SO_2_, and metal ions, in modulating the formation of H_2_S post-bottling was demonstrated. When present in equimolar concentrations a 1:1 ratio of H_2_S- and SO_2_-catechol adducts were produced. At wine relevant concentrations, however, only SO_2_-adducts were produced, reinforcing that the competition reactions of sulfur nucleophiles, such as H_2_S and SO_2_, with wine matrix compounds play a critical role in modulating final H_2_S concentrations in wines.

## 1. Introduction

The origin and management of volatile sulfur compounds (VSCs) in wine is a topic that concerns many winemakers, as these compounds have a significant impact on wine aroma as well as wine quality [1]. Volatile sulfur compounds such as hydrogen sulfide (H_2_S) contribute negatively to wine quality and are considered a wine fault when present at concentrations greater than its odor threshold (OT) value of 1.1–1.6 μg/L [2]. As one of the main compounds contributing to “reduced” characters in wine, H_2_S imparts aromas of rotten egg and sewage when present in high concentrations, however, when present in low concentrations H_2_S can add complexity to wine aroma [2]. While the release and subsequent perception of H_2_S into the wine headspace is affected by the wine matrix, the contribution of individual wine matrix components on VSC perception, such as H_2_S, has not yet been determined [3].

The reaction pathways for H_2_S formation post-bottling are not yet as well defined as those during fermentation, however, certain key factors affecting H_2_S concentration post-bottling have recently been elucidated. It is known that decreased oxygen concentrations during fermentation as well as during wine storage conditions are associated with increased H_2_S concentrations in wines post-bottling [4,5,6]. Franco-Luesma and Ferreira [7] have recently shown that H_2_S is present in both free and bound forms in wine and the percentage of free and bound forms are related to the trace metal content of the wine. According to Franco-Luesma and Ferreira, both de novo formation of H_2_S from precursor compounds as well as the release of H_2_S from metal complexes contribute to the final concentration of H_2_S formation in wines post-bottling, with release from metal complexes responsible for the majority of H_2_S produced in red wines, and de novo formation responsible for the majority of H_2_S produced in white wines and rosé wines [7]. Additionally, Franco-Luesma suggests that the release of free H_2_S and MeSH from bound sources are a function of a decrease in redox potential of wine [8].

A number of studies have also highlighted the important catalytic role of metals such as Cu^2+^, Fe^3+^, Mn^2+^, Al^3+^, and Zn^2+^ ions in VSC formation in wine [4,9,10,11]. Metal ions naturally occur in wines at trace concentrations and their final concentrations in finished wine are influenced by the use of metal containing pesticides and herbicides, processing aids, and winemaking equipment. However, the addition of Cu^2+^ (copper can exist in both Cu^+^ and Cu^2+^ oxidation states in wine; thus, in this manuscript, “Cu^2+^” is used when the authors refer to the oxidation state in which copper was added to the wine) to wines through the use of copper sulfate as a fining agent applied to decrease reductive aromas in wine, remains the main use of metals that results in increased residual concentration of the metal post-bottling [5]. While the mechanism of copper fining is not yet well defined, Kreitman et al. [12] recently provided insights into the mechanisms through which Cu^2+^ interacts with thiols such as H_2_S in wine-like conditions saturated with oxygen. The manner in which residual copper sulfide (CuS) may affect wine aroma has recently been studied by Clark et al., who firstly showed that once formed, CuS is not easily removed from white wine and that tartaric acid significantly affects CuS precipitation [13]; and secondly, that in both model and finished wines CuS was not measurable as labile copper but that oxidation as well as the loss of volatile thiol compounds through volatilization affects the ratio of labile copper to CuS [14]. These studies demonstrated that when copper and H_2_S are present in wines, non-labile CuS will be produced that cannot be removed through filtering and that changes in the wine conditions will affect the ability of copper to remain active/labile to mediate reactions. The ability of copper to mediate H_2_S formation reactions have been shown in wines where the addition of Cu^2+^ was associated with significantly increased H_2_S concentration in wines during bottle aging [4,10,15]. Given that a major source of Cu^2+^ in finished wines is within the control of the winemaker, the timing and amount of Cu^2+^ can be used to manage H_2_S formation.

Another important winemaking intervention is the use of sulfur dioxide (SO_2_). The dosage and timing of SO_2_ addition are similarly key activities under winemaking control and may impact H_2_S formation. Sulfur dioxide naturally occurs in wines at very low concentrations (10–30 mg/L [16]), however, the main contributor to the final concentration in commercial wines is added SO_2_ at concentrations in the range of 50–200 mg/L. Sulfur dioxide exists in free and bound forms, with the majority of free SO_2_ present as bisulfite ions (HSO_3_^−^) at normal wine pH (all of the different species of sulfur dioxide in equilibrium found in wine, which includes the molecular sulfur dioxide, bisulfite, and sulfite, will be referred to generically as “SO_2_” throughout the text). Sulfur dioxide plays a critical role in the prevention of microbial spoilage, and is important as an antioxidant, through reaction with hydrogen peroxide (H_2_O_2_) generated from oxygen via a series of redox steps, as well as with quinones to regenerate polyphenols [1,17,18,19,20]. It is also known that SO_2_ influences the loss of thiol compounds post-bottling [21,22]. It has also been proposed that SO_2_ may act as a source of VSCs. Rankine suggested the reduction of SO_2_ through the interaction of metal ions such as manganese and zinc with tartaric and malic acids during fermentation [23]. No evidence for the post-bottling formation of H_2_S via the metal catalyzed reduction of SO_4_^2−^ or SO_3_^2−^ during low oxygen conditions has yet been shown, even though this pathway has been proposed by Ribéreau-Gayon [24] and Lopes et al. [25]. Another possible pathway through which SO_2_ can be involved in the modulation of “reductive” VSCs post-bottling is through reactions with wine compounds, such as quinones. Nikolantonaki et al. [26] described the reaction and kinetics between certain o-quinones and nucleophilic thiols and has shown that the addition of SO_2_ directly influenced the rates and the yields of all of the o-quinone sulfur adducts. Research has demonstrated, using model wine studies, that sulfites, ascorbic acid, and/or GSH can act as sacrificial nucleophiles, suppressing varietal thiol consumption during wine aging [21,22]. This competing reaction between H_2_S, methanethiol (MeSH), glutathione, and SO_2_ has not yet been demonstrated in real wines.

The separate effects that Cu^2+^ and SO_2_ additions may have on H_2_S formation in wines post-bottling have been discussed above, however, Cu^2+^ and SO_2_ are usually both present in wine and may have synergistic effects via wine matrix compounds. Given that the final concentrations of Cu^2+^ and SO_2_ in finished wines, as well as timing of the addition of Cu^2+^ and SO_2_, are under a winemaker’s control it is important to understand the synergistic interactions between wine additives such as Cu^2+^ and SO_2_ and the effects that these compounds may have on H_2_S formation. The interactions between Cu^2+^ and SO_2_ and its effects on H_2_S formation have not previously been researched in a structured way in real wines, and this synergistic “Cu^2+^ + SO_2_” interaction may be an additional key contributing factor in the accumulation of H_2_S post-bottling. The aim of the present study was to investigate the synergistic effects of the combined treatment of Cu^2+^ and SO_2_ on H_2_S formation in a white wine (Verdelho) and a red wine (Shiraz). Some of the factors modulating the formation of H_2_S via Cu^2+^ and SO_2_ interactions during wine storage under low oxygen conditions were also studied.

## 2. Results

### 2.1. Dissolved Oxygen

The dissolved oxygen (DO) was monitored over the course of the experiment using PreSens oxygen sensors applied to representative colorless vials (in triplicate) for a control sample (blank wine) and for each of the treatments (Cu^2+^, SO_2_, and “Cu^2+^ + SO_2_”) for the Verdelho (*n* = 12) and for the Shiraz (*n* = 12) wine samples. After two months of storage under low oxygen conditions, both Verdelho and Shiraz samples had consumed all available oxygen and samples can be considered oxygen-free. All samples were stored at room temperature (22 °C) in 19 L post mix Cornelius kegs (Ambar technology, Alexandria, NSW, Australia). The kegs were flushed with nitrogen gas (N_2 (g)_) to <1 ppb and maintained at a slight positive pressure of 1 psi N_2 (g)_ to prevent any oxygen ingress into the keg storage systems, as described by Viviers et al. [10]. As such all wine samples were not exposed to oxygen during storage and only exposed to normal atmospheric oxygen at the point of analysis.

### 2.2. Formation of Hydrogen Sulfide in Wine

#### 2.2.1. The Effect of Cu^2+^ on H_2_S Formation

**Figure 1:**
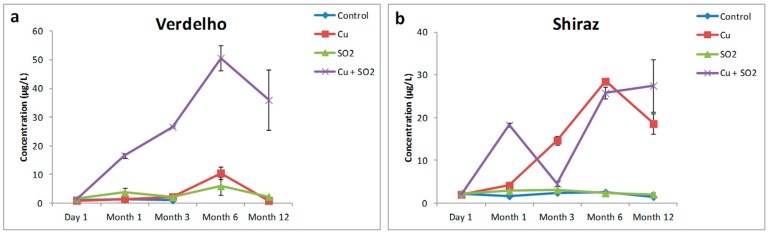
The effects of Cu^2+^, SO_2_, and “Cu^2+^ + SO_2_” on the formation of H_2_S in (**a**) Verdelho and (**b**) Shiraz wine samples that were stored under nitrogen (N_2_) and measured over the course of 12 months.

Copper treatment did not result in an overall significant effect on H_2_S formation in the Verdelho samples. Significant increases in H_2_S concentration were measured after six months of anaerobic storage associated with Cu^2+^ treatment, however, the overall Cu^2+^ effect, as measured over the course of the twelve months, was not significant for H_2_S formation (Figure 1). Hydrogen sulfide is a reactive nucleophile and it has been shown to react readily with quinones in wine-like solutions [21] which may explain the decrease in H_2_S concentration after the initial increase measured after six months of storage. Only in the Shiraz wine samples did Cu^2+^ treatment alone result in an overall significant effect on H_2_S formation. All Shiraz wine samples treated with Cu^2+^ displayed significant increases in H_2_S concentration, irrespective of the other additional treatments (Figure 1b, Table 1). Multi-way analysis of variance (ANOVA) confirmed that the increased H_2_S concentration was significantly correlated with the Cu^2+^ addition and not associated with interactions between Cu^2+^ and “SO_2_” in the “Cu^2+^ + SO_2_” treatment.

The effect of residual Cu^2+^ on H_2_S formation is well established [4,10,27]. All the precursors to H_2_S have not yet been identified, however, wines with elevated residual Cu^2+^ concentrations post-bottling risk the formation of H_2_S during bottle maturation.

#### 2.2.2. The Effect of SO_2_ on H_2_S Formation

The treatment of both Verdelho and Shiraz wine samples with SO_2_ alone had no significant effect on H_2_S concentration (Figure 1a,b). No increased H_2_S concentrations were measured by treating Verdelho or Shiraz wines with SO_2_ alone, suggesting that for the wines investigated in the current study, SO_2_ is not capable of significantly increasing H_2_S concentrations when present in wines without Cu^2+^.

#### 2.2.3. The Effects of Cu^2+^ and SO_2_ Interaction on H_2_S Formation

The combined Cu^2+^ and SO_2_ treatment in the Verdelho wine samples resulted in significant and large increases in H_2_S concentrations. For the Verdelho samples more H_2_S was measured in samples treated with “Cu^2+^ + SO_2_” than by treating samples with Cu^2+^ alone (Figure 1a). Using multi-way ANOVA it was possible to separate the effects of Cu^2+^ treatment from the combined Cu^2+^ and SO_2_ effects, with the increase in H_2_S concentration significantly associated with “Cu^2+^ + SO_2_” treatment in the Verdelho samples (*p* < 0.001, Table 1). The concentration of H_2_S produced in Verdelho samples treated with “Cu^2+^ + SO_2_” was 36.01 (±18.1) µg/L compared to the 0.879 (±0.028) µg/L of H_2_S measured in the control samples after 12 months of storage under low oxygen conditions, which is a significant increase in H_2_S concentration.

Significantly increased H_2_S concentrations were also measured in the Shiraz samples treated with “Cu^2+^ + SO_2_” (Figure 1b). By making use of multi-way ANOVA it was clear that these increases were only associated with a significant effect of Cu^2+^ (*p* < 0.001, Table 1) and not associated with the combined Cu^2+^ and SO_2_ treatments (Table 1, Figure 1b). If the evolution of H_2_S in Shiraz samples is considered over the course of the 12 months of storage in a low oxygen environment, the trends of H_2_S formation for Cu^2+^ treated samples and samples treated with both Cu^2+^ and SO_2_ are remarkably different (Figure 1b). Shiraz samples treated with only Cu^2+^ displayed a steady increase in H_2_S concentrations from Month 1 to Month 6, followed by a decrease to Month 12, whereas samples treated with Cu^2+^ and SO_2_ displayed an initial increase from Day 0 to Month 1, followed by a significant decrease in H_2_S concentrations at Month 3, which was then followed by increased H_2_S concentrations from Month 6 through to Month 12. The exact mechanism for these different H_2_S evolution trends are not clear, however, this demonstrates the complex underlying interactions of different wine compounds with one another and how the addition of SO_2_ to a wine already containing Cu^2+^ could have significant implications on H_2_S evolution. Previous studies have also shown the non-linear increase in thiols such as H_2_S and MeSH evolution over time [4,5,10], which suggest that compounds such as H_2_S and MeSH may be dynamically bound and released by other wine compounds after bottling and during storage.

### 2.3. Interaction of Cu^2+^ and SO_2_ in Model Wine

#### 2.3.1. Formation of H_2_S in Model Wine

**Figure 2:**
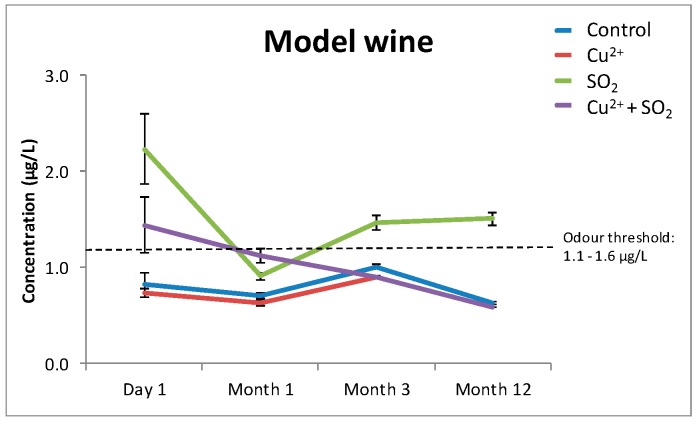
The effects of Cu^2+^, SO_2_, and “Cu^2+^ + SO_2_” on the concentration of H_2_S in a model wine matrix that did not contain any wine compounds, such as polyphenols or tannins, that could modulate the formation of H_2_S. The odor threshold of H_2_S (1.1–1.6 µg/L [28]) is indicated with the dashed line parallel to the *x*-axis. Samples were stored under nitrogen (N_2_) and measured over the course of 12 months.

In an effort to identify some of the potential mechanisms involved in modulating the significantly increased H_2_S concentrations in Verdelho samples treated with “Cu^2+^ + SO_2_”, the experiment was repeated in a model wine containing only water, ethanol (12%), and tartaric acid (10 g/L). Samples treated with “Cu^2+^ + SO_2_” did not produce similar significant increases in H_2_S concentrations as was measured in the Verdelho samples treated with “Cu^2+^ + SO_2_” (Figure 2). While there were some changes in H_2_S concentrations in SO_2_ treated samples (Figure 2), the increases were small compared to the significantly increased H_2_S concentrations measured in Verdelho samples treated with “Cu^2+^ + SO_2_” (Figure 1a).

From this experiment, it is clear the increases measured in H_2_S associated with “Cu^2+^ + SO_2_” treatment require other wine matrix components.

#### 2.3.2. Reactions between H_2_S and SO_2_ with a Model Wine Compound/Thiol Quenching Compound

It is known that certain wine matrix compounds, such as quinones, for example, have the ability to readily react with thiols in wine [21] and thus play an important role in modulating the final concentrations of thiols in wines. To investigate the competition reactions between H_2_S and SO_2_ with wine matrix compounds capable of reacting with thiols, a model quinone (4-methylbenzoquinone (4MBQ)) was synthesized and H_2_S and SO_2_ were added to 4MBQ to produce adducts of 4MBQ with H_2_S and SO_2_. The sulfur containing nucleophiles were first reacted in excess concentration (4 mM) relative to 4MBQ (1 mM) and the reaction products tentatively identified using high pressure liquid chromatography (HPLC), liquid chromatography-mass spectrometry (LCMS), liquid chromatography-high resolution mass spectrometry (LC-HRMS), and comparison of the mass spectra with published results [21]. All the isomers produced as reaction products from reacting 4MBQ with H_2_S will from here on be referred to as “H_2_S-4MC” adducts. Similarly, all the isomers produced as reaction products from reacting 4MBQ with SO_2_ will from here on be referred to as “SO_2_-4MC”. Synthesized H_2_S-4MC and SO_2_-4MC adducts were used as standards for retention time. The main reaction products are listed in Table 2. The high reactivity of the SO_2_ and H_2_S in relation to the 4MBQ resulted in a high number of isomeric compounds being produced, and for the purpose of this study, only the major isomeric compounds were considered.

##### Competition Reactions between H_2_S and SO_2_ with a Model Wine Quinone

**Figure 3:**
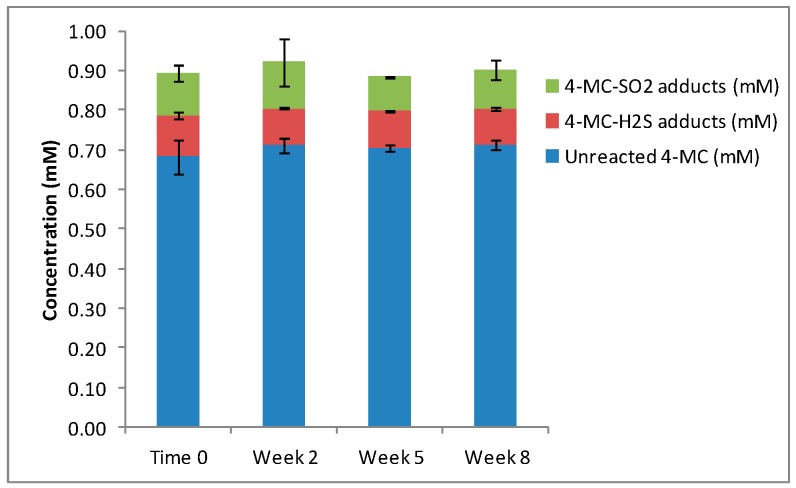
The reaction products measured when 4MBQ was added to model wine containing equimolar concentrations of H_2_S and SO_2_.

When 4-methylcatechol (4MC) was oxidized to 4MBQ, the HPLC and LCMS analyses showed only approximately 20% of the 4MBQ reacted with the nucleophiles, while the remaining 4MBQ fraction was converted back to 4MC. This incomplete conversion of 4MC to 4MBQ is agreement with previous studies [21]. When 4MBQ (1 mM) was incubated with H_2_S (4 mM), three main isomers were formed at a ratio of 5:3:1, and when 4MBQ (1 mM) was incubated with SO_2_ (4 mM), four main isomers were formed at a ratio of 2:1:3:1 (Table 2). When 4MBQ (1 mM) was added to model wine containing both sulfur nucleophiles in equimolar concentrations of 2 mM respectively, the same concentration of H_2_S-4MC and SO_2_-4MC adducts were measured (Figure 3), with a peak ratio of 4:1:3:1 for H_2_S-4MC and the three main SO_2_-4MC adducts. There were no statistical differences in the total concentrations of H_2_S-4MC and SO_2_-4MC adducts produced (*p* = 0.193). The distribution of the H_2_S-4MC and SO_2_-4MC adducts remain approximately the same throughout the eight weeks of the experiment (Figure 3).

This experiment demonstrated that when H_2_S and SO_2_ are present in equimolar concentrations, both sulfur nucleophiles compete equally for binding to the quinone and a 1:1 ratio of H_2_S-4MC and SO_2_-4MC adducts are produced.

##### Competition Reactions between a Model Wine Quinone with H_2_S and SO_2_ at Wine Relevant Concentrations

**Figure 4:**
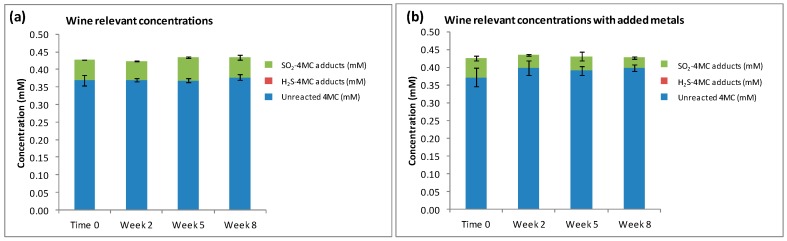
The reaction products measured when 4MBQ (0.45 mM) was added to model wine containing: (**a**) wine relevant concentrations of H_2_S (0.39 µM) and SO_2_ (0.23 mM); and (**b**) wine relevant concentrations of H_2_S (0.39 µM), SO_2_ (0.23 mM), as well as Cu^2+^ (0.5 mg/L) and Fe^3+^ (4.0 mg/L).

When 4MC was oxidized to 4MBQ, and added to model wine containing H_2_S and SO_2_ at wine relevant concentrations (4MC, 0.45 mM; H_2_S, 0.39 µM; SO_2_, 0.23 mM), SO_2_ preferentially reacted with 4MBQ to form a mixture of SO_2_-4MC adducts (Figure 4a) even though the spiked H_2_S was available in the sample to react with 4MBQ (see H_2_S concentration data in Figure S1). The peak ratio for the three main SO_2_-4MC adducts was 1:5:1. Considering that H_2_S and SO_2_ have approximately identical first order rate constants when reacting with 4MBQ [21], it was anticipated that the SO_2_-4MC adducts would be the main 4-MC adducts produced, but this experiment confirmed that it was indeed the case. This experiment demonstrated that when H_2_S and SO_2_ are present in wine relevant concentrations of 0.39 µM H_2_S and 0.23 mM SO_2_, the SO_2_-4MC adducts dominate and no H_2_S-4MC adducts can be measured. This suggests that thiol quenching compounds may fully react with sulfur nucleophiles that are present in higher concentrations, such as SO_2_, and thus allow the accumulation of sulfur nucleophiles present in lower concentrations, such as H_2_S, for example.

The concentrations of SO_2_-4MC adducts measured in samples with added Cu^2+^ and Fe^3+^ were significantly decreased when compared with the samples without any added metal ions (*p* = 0.019) (Figure 4b), and a peak ratio of 1:2:1 was measured for the three main SO_2_-4MC adducts. This decrease in SO_2_-4MC adducts may be attributed to the interaction between SO_2_ and the metal ions when reacting with oxygen as described by Danilewicz [29]. No H_2_S-4MC adducts were measured in samples with added metals, even though a small concentration of H_2_S was measured at Time 0 (Figure S1). No H_2_S was observed in the samples post Time 0, which may be attributed to the reaction between Cu^2+^ and H_2_S, a reaction through which CuS complexes are produced, preventing H_2_S from reacting with the quinone to produce H_2_S-4MC adducts.

## 3. Discussion

### 3.1. Impact of Oxygen on the Formation of H_2_S from Cu^2+^ + SO_2_ Interaction in This Study

DO was monitored over the course of the experiment using PreSens oxygen sensors applied to representative colorless vials (in triplicate) for a control sample (blank wine) and for each of the treatments (Cu^2+^, SO_2_, and “Cu^2+^ + SO_2_”) for the Verdelho (*n* = 12) and for the Shiraz (*n* = 12) wine samples. The rate of oxygen consumption varied between the Verdelho and Shiraz wine samples, with the Shiraz samples consuming the available oxygen faster than the Verdelho samples. The rate of oxygen consumption also varied between the Cu^2+^ and SO_2_ treated wine samples (Figure S2). Verdelho samples treated with SO_2_ displayed the fastest rate of oxygen consumption, and samples treated with Cu^2+^ also displayed a faster oxygen consumption rate than samples without any added Cu^2+^ (Figure S2a). The interaction between oxygen and SO_2_ in wine, the differences in oxygen consumption rates between red and white wine, and the effect of metals on the oxygen consumption rate in wine is in agreement with trends observed by other authors [17,19,24,29]. After two months of storage under low oxygen conditions, both Verdelho and Shiraz samples had consumed all available oxygen and samples can be considered to be effectively oxygen-free (Figure S2a,b).

### 3.2. Formation of H_2_S Associated with Cu^2+^ + SO_2_ Treatment

The role of the interactive effects of residual Cu^2+^ and free SO_2_ in the modulation of H_2_S concentrations in wines post-bottling has not previously been considered. A significant source of Cu^2+^ in wines is through intentional addition during copper fining remediation treatments, and the dosage and the timing of these Cu^2+^ additions are determined by the winemaker. Similarly, SO_2_ can be added pre-fermentation, post-fermentation, during maturation and before bottling. In this study, wines were not previously treated with Cu^2+^ or SO_2_. The first additions of Cu^2+^ and SO_2_ for the Verdelho and Shiraz wines represented an “at-bottling” treatment for both Cu^2+^ and SO_2_. In the Verdelho samples, treatment with “Cu^2+^ + SO_2_” significantly increased H_2_S concentrations after 12 months of anaerobic storage. Concentrations of up to 36.01 (±18.1) µg/L H_2_S were measured in the “Cu^2+^ + SO_2_” treated Verdelho samples compared to the 0.879 (±0.028) µg/L of H_2_S in the control samples. This study has demonstrated that in wines produced without early Cu^2+^ and SO_2_ additions, but treated with these additives before bottling may increase the risk of developing elevated H_2_S concentrations post-bottling. The high concentration of free SO_2_ available due to late SO_2_ treatment just before bottling may explain why the interactive effects of Cu^2+^ and SO_2_ were associated with such significant increased H_2_S concentrations in Verdelho wines.

**Scheme 1:**
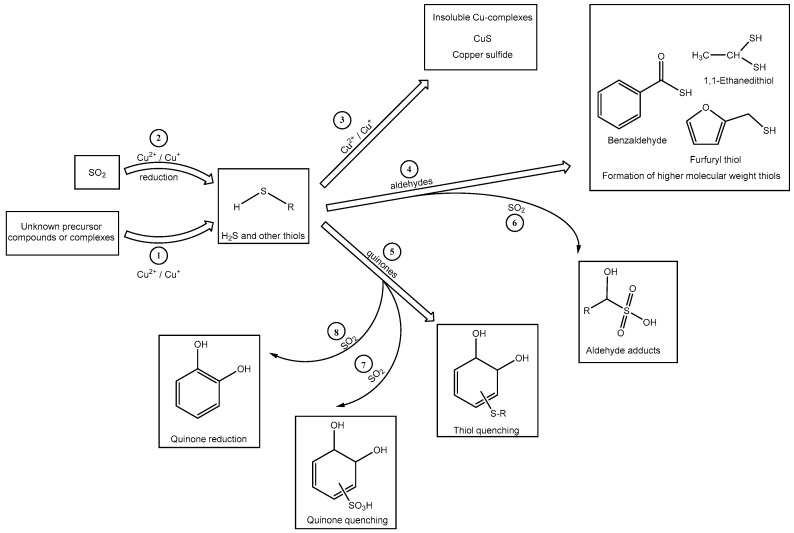
Possible mechanisms for the formation of H_2_S and other low molecular weight thiol compounds and the interaction between Cu^2+^, SO_2_, and “Cu^2+^ + SO_2_” with wine compounds such as aldehydes and o-quinones, during H_2_S formation.

The exact mechanism for the formation of H_2_S due to the treatment of Verdelho samples with “Cu^2+^ + SO_2_” is not yet known (Figure 1). It is possible that in the samples spiked with “Cu^2+^ and SO_2_”, H_2_S is produced through a Cu^2+^ mediated reaction (Scheme 1, (1)) from unknown precursors naturally present in the wine, either as complexes or in molecular precursor forms. The produced H_2_S can be quenched through addition to either wine quinones or aldehydes [Scheme 1, (4) and (5)]. However, when a high concentration of free SO_2_ is available, competing reactions between SO_2_ and H_2_S for quinones/aldehydes may occur (Scheme 1, (6), (7), and (8)). It is known that nucleophilic addition reactions compete with the two electron reduction of quinones by SO_2_ [22]. The first-order rate constants for the addition of SO_2_ or H_2_S to quinones are practically identical [21], thus the concentration of these nucleophiles determines the preferential addition of SO_2_ or H_2_S to quinone molecules. Given that SO_2_ was added in mM concentrations, whereas H_2_S was produced in µM concentrations, the addition of SO_2_ to quinone molecules or the reduction of the quinones via SO_2_ may be the favored reaction (Scheme 1, (7)). The reaction of bisulfite with wine quinones to produce the sulfonic acid derivative or that reduces the quinone back to its corresponding diphenolic compound [30], may prevent the quenching of thiols and thereby indirectly increase H_2_S concentrations in the Verdelho samples. Another possible quenching reaction could be through the addition of H_2_S to wine carbonyl compounds such as acetaldehyde, a compound known to readily react with thiols [31]. The availability of free SO_2_ and the formation of H_2_S through Cu^2+^ mediated reactions would lead to competition reactions between these two sulfur containing molecules for the available carbonyl compound (Scheme 1, (4)), which could result in a higher concentration of H_2_S in samples treated with SO_2_ than in treatments without SO_2_. Another explanation may be the suggested reduction of SO_2_ by Cu^2+^ that results in the direct formation of H_2_S (Scheme 1, (2)), a proposed mechanism for wine post-bottling by Ribéreau-Gayon and Lopes et al. [24,25].

The benefits of treating wine with SO_2_ offsets the risk of developing “reduced” aromas, and this study demonstrated that even late SO_2_ additions close to bottling did not pose a risk of increased H_2_S unless there was a high concentration of residual Cu^2+^ present in the wines. This study emphasizes the “Cu^2+^ + SO_2_” interaction effect and that increased free SO_2_ concentrations combined with elevated residual Cu^2+^ concentrations in wines post-bottling poses a risk of increasing H_2_S concentrations post-bottling. As such it is important to ensure that residual Cu^2+^ concentrations, which can be controlled by the winemaker, are kept as low as possible in finished wine.

In the Shiraz samples treated with “Cu^2+^ + SO_2_” the significant increases in H_2_S concentrations were associated with Cu^2+^ treatment (*p* < 0.001, Table 1) and not associated with the combined Cu^2+^ and SO_2_ treatments (Table 1, Figure 1b). It is important to note that the Shiraz wine had approximately 50% less free SO_2_ than the Verdelho wine, and in red wine a significant proportion of the free SO_2_ is loosely bound to anthocyanins and other phenolic compounds [17] and as such may not be as available for participation in reactions associated with increased H_2_S concentrations. In the Verdelho wine samples, a higher concentration of free SO_2_ was available to be involved in reactions associated with the increase in H_2_S. This difference in free SO_2_ concentrations may have contributed to the varying effects of Cu^2+^ and “Cu^2+^ + SO_2_” measured in these two red and white wines. A previous report [10] also showed the varying effect of Cu^2+^ on H_2_S formation in Chardonnay relative to Shiraz wines and demonstrated that H_2_S increased in Shiraz samples spiked with Cu^2+^ when stored in a low oxygen environment, but not in Chardonnay samples treated in the same way. The opposing Cu^2+^ effects seen in this study for the Verdelho wine samples relative to Shiraz wine samples again illustrates the complexity of the reactions taking place in wines with different matrices.

### 3.3. Mechanisms Modulating H_2_S Formation

The experiments described in Section 2.3.1 where the “Cu^2+^ + SO_2_” treatment was applied to model wine demonstrated that no H_2_S was produced in model wine containing only water, EtOH, tartaric acid, Cu^2+^, and SO_2_. This shows that other wine matrix compounds were necessary to produce the significantly increased H_2_S concentrations measured in the Verdelho samples treated with “Cu^2+^ + SO_2_”. The increased H_2_S concentration measured in Verdelho samples treated with both “Cu^2+^ + SO_2_” may be the result of the interaction of wine compounds such as wine quinones, aldehydes or any other wine compounds that possess the ability to react with H_2_S.

The quinone (4MBQ) of a representative wine polyphenol (4MC) was added to model wine and the competition reactions between H_2_S and SO_2_ were studied at equimolar concentrations as well as at wine relevant concentrations of H_2_S and SO_2_. It is known that H_2_S and SO_2_ have similar first order rate constants [21] and when H_2_S and SO_2_ were added at wine relevant concentrations with SO_2_ concentrations significantly greater than H_2_S concentrations, the SO_2_-quinone adducts were the major reaction product produced. This experiments showed that concentrations in which sulfur nucleophiles are present in wine would determine the major quinone-adduct produced in the case of sulfur nucleophiles with similar first order rate constants. When H_2_S and SO_2_ are involved in such competition reactions at wine relevant concentrations, fewer H_2_S molecules will be quenched through reactions with quinones, leading to an indirect increase in H_2_S concentrations (Scheme 1 (5) and (7)). These results suggest that the effects of Cu^2+^ and SO_2_ may operate independently on separate pathways, but the effects of the additives are cumulative, leading to an increased H_2_S concentration.

This study has shown that the presence of wine additives, such as Cu^2+^ and SO_2_, play a fundamental role in the modulation of H_2_S profiles in both red and white wine post-bottling. Although the exact reaction pathway for the formation of H_2_S associated with the combination of Cu^2+^ and SO_2_ is not yet known, there are a few possible mechanisms. When a higher ratio of free SO_2_ to H_2_S is available, it can form adducts with wine quinones or reduce them back to their corresponding phenols, and in doing so prevent the quenching of the newly formed thiols. Competing reactions between H_2_S and SO_2_ could also result in a smaller percentage of H_2_S lost through, for example, reactions with acetaldehyde. This could indirectly lead to a relative increase in thiol concentration in the treatments with a high concentration of carbonyl compounds. Another explanation may be the suggested reduction of SO_2_ by Cu^2+^ that might lead to the formation of H_2_S, as proposed by Ribéreau-Gayon [24] and Lopes et al. [25], although studies in our model wine have not supported this.

Recent work by Franco-Luesma and Ferreira proposed two methods to access H_2_S production from “loosely bound” vs. “de novo” formation [32]. In this study a method that is comparable with the “total” H_2_S quantification method was applied that relied on fully releasing all the H_2_S into the wine headspace through the addition of 2 g of salt into 10 mL of wine [28]. As such the H_2_S loosely bound were fully released, measured and quantified in all samples at the first measurement at Day 0. The same method was used to analyze the samples over a 12-month period and increased H_2_S concentrations were statistically significantly associated with the combined “Cu^2+^ + SO_2_” treatment and H_2_S concentration was shown to increase over time in samples, indicating de novo formation of H_2_S associated with “Cu^2+^ + SO_2_” treatment.

The interactive effects of Cu^2+^ and SO_2_ have not previously been explored as a significant factor associated with the accumulation of H_2_S in wines post-bottling. The majority of commercial wines are treated with SO_2_ and many contain Cu^2+^ incorporated through uptake from the soil, pickup during the winemaking process, or by direct addition of Cu^2+^ through remediation treatments later in production. Fully understanding the timing effects of early and/or late SO_2_ and Cu^2+^ additions will help determine whether a wine could be placed at risk of developing elevated H_2_S concentrations later during storage.

## 4. Materials and Methods

### 4.1. Materials

Amberlyst A26 hydroxide form, ethanol (99.5%), ethylmethyl sulfide (EMS, 96%), iron (III) sulfate hydrate (97%), 4-methylcatechol (4MC, 95%), periodic acid (99%), potassium hydrogen tartrate (99%), potassium metabisulfite (PMS, 98%), and sodium hydrosulfide hydrate (NaSH·xH_2_O, 71%) were obtained from Sigma-Aldrich (Castle Hill, NSW, Australia). Propyl thioacetate (99.7%) was obtained from Lancaster Synthesis (Jomar Bioscience, Kensington, SA, Australia). Acetonitrile (ACN, gradient grade for liquid chromatography), methanol (MeOH, 99.8% by GC), ortho-phosphoric acid (85%), tartaric acid, tetrahydrofuran (THF), and sodium chloride were obtained from Merck (Frenchs Forest, NSW, Australia). Water was obtained from a Milli-Q purification system (Millipore, North Ryde, NSW, Australia). Copper (II) sulfate pentahydrate (99%) was purchased from Ajax Chemicals (Sydney, NSW, Australia). Formic acid (98%–100%) was purchased from Rowe Scientific (Lonsdale, SA, Australia).

### 4.2. Wine Samples

Certified organic Verdelho wine from the 2012 vintage and Shiraz wine from the 2010 vintage, produced in South Eastern Australia, were obtained from a local winery. Neither wine was treated with either SO_2_ or Cu^2+^ during winemaking. Analyses of the chemical compositions of the two base wines were conducted by The Australian Wine Research Institute (AWRI) Analytical Service (ISO 17025 Laboratory, Adelaide, Australia) and are as follows: pH 3.33, 5.8 g/L residual sugars,12.63% (*v*/*v*) alcohol, 0.85 g/L volatile acidity (as acetic acid), 6.1 g/L titratable acidity (as tartaric acid), <4 mg/L free SO_2_ and <4 mg/L total SO_2_ for the Verdelho wine; and pH 3.46, 0.7 g/L residual sugars, 14.0% (*v*/*v*) alcohol, 0.37 g/L volatile acidity (as acetic acid), 6.6 g/L titratable acidity (as tartaric acid), <4 mg/L free SO_2_ and <4 mg/L total SO_2_ for the Shiraz wine.

### 4.3. Chemical Analyses

#### 4.3.1. Oxygen Measurement

Colorless 20 mL crimp top vials (Chromacol, Part of Thermo Fisher Scientific Inc., Scoresby, VIC, Australia) were fitted with PreSens Pst6 oxygen sensors (Presens, Regensburg, Germany) and filled with the three treatments (Cu^2+^, SO_2_, and “Cu^2+^ + SO_2_”) and control samples (without any treatments) in triplicate for the Verdelho (*n* = 12) and Shiraz wine (*n* = 12) and were used to measure DO during storage of the wines. Samples were stored at 20 °C under light free conditions and under nitrogen (N_2_) as described by Viviers et al. [10]. Oxygen measurements were carried out using a PreSens Fibox 3 trace v3 oxygen meter, with the limit of detection when using a PtS6 sensor of 1 ppb oxygen. (Presens, Regensburg, Germany).

#### 4.3.2. Metal Quantifications

Base wines were analyzed for their metal concentrations by Flinders Analytical, Flinders University (Adelaide, Australia) using an Agilent 7500 cx inductively coupled plasma mass spectrometers (Agilent Technologies, Tokyo, Japan) as described in Thiel et al. [33]. Stock solutions of Cu^2+^ were prepared volumetrically in an anaerobic hood using degassed water (Milli-Q), and then measured for Cu^2+^ concentrations. The concentrations of total Cu^2+^ in the base Verdelho and Shiraz wines, as well as the spiked total Cu^2+^ concentrations in the Verdelho and Shiraz wines are given in Table 3. The stock solution of Fe^3+^ was prepared as for Cu^2^.

#### 4.3.3. Determination of SO_2_

Base wines and the SO_2_ stock solution prepared for addition to the samples were analyzed for their free and total SO_2_ concentrations using the Lachat flow injection analysis (FIA) system by the AWRI Analytical Service (ISO 17025 Laboratory, Adelaide, Australia). Fresh stock solutions of SO_2_ were prepared volumetrically in an anaerobic hood by dissolving PMS in degassed water (Milli-Q) on the day of the SO_2_ addition and discarded after use. The concentrations of SO_2_ in the base Verdelho and Shiraz wines, as well as the spiked SO_2_ concentrations in the Verdelho, Shiraz, and model wines are given in Table 3.

#### 4.3.4. Preparation of Reaction Products of H_2_S and SO_2_ with 4-Methylbenzoquinone

The preparation of 4MBQ by periodate resin was as described by Jongberg et al. [37] and Nikolantonaki et al. [21], with 4MBQ used immediately after synthesis. The adducts of SO_2_ and H_2_S, used for retention time reference standards and for the comparison of mass spectral data, were synthesized as follows: 4MBQ (1.1 mM) was prepared as described by Nikolantonaki et al. [21] and dissolved in cold (4 °C) model wine (12% EtOH, 10 g/L tartaric acid, pH 3.4) containing SO_2_ (4 mM) or H_2_S (4 mM). The reaction mixture was stirred at 20 °C for 15 min. All reaction products were characterized using LC-HRMS (Table 2), as well as comparing their mass spectra with published spectral data [21].

The competition reaction between SO_2_ and H_2_S with 4MBQ was studied in three separate experiments. First, synthesized 4MBQ (1 mM) was added to cold (4 °C) model wine (12% EtOH, 10 g/L tartaric acid, pH 3.4) that already contained SO_2_ (2 mM) and H_2_S (2 mM) at equimolar concentrations. The second and third experiments were conducted at wine relevant concentrations, where 4MBQ (0.45 mM) [38,39] was added to cold (4 °C) model wine (12% EtOH, 10 g/L tartaric acid, pH 3.4) that already contained either (2) SO_2_ (0.23 mM) [36] and H_2_S (0.39 µM) [28]; or (3) SO_2_ (0.23 mM) [36] and H_2_S (0.39 µM) [28] with added Cu^2+^ (0.5 mg/L) [34] and Fe^3+^ (4.0 mg/L) [34]. In all three experiments, the reaction mixtures were stirred at 20 °C for 15 min. The reaction between the nucleophiles and 4MBQ was followed using HPLC. Additionally, residual H_2_S and SO_2_ concentrations were measured using GC-SCD and FIA, respectively.

#### 4.3.5. Analysis of 4-Methylbenzoquinone Adducts Using Liquid Chromatography

The reaction between 4MBQ with SO_2_ and H_2_S was monitored and the reaction products quantified using the high pressure liquid chromatography (HPLC) as described in Mercurio et al. [40] with slight modification. Briefly, samples were analyzed using an Agilent 1100 LC (Agilent, Mulgrave, VIC, Australia) with a Phenomenex Synergi Hydro-RP C18 column (150 mm × 2 mm, 4 μm) at 25 °C. Solvent A consisted of 1% ACN and 1.5% phosphoric acid in H_2_O, and solvent B was 80:20 ACN/Solvent A, for gradient elution at a flow rate of 0.4 mL/min: 0 min (0% solvent B), 10 min (5% solvent B), 15 min (20% solvent B), 17 min (100% solvent B), and 30 min (0% solvent B).

The reaction products of 4MBQ with SO_2_ and H_2_S were quantified using LCMS as well as LC-HRMS. An Agilent 1200 HPLC system (Forest Hill, Victoria, Australia) equipped with binary pump (1290 model), degasser, autosampler, column oven and diode array detector (DAD) was used. A 5 µL aliquot of a sample was injected and separated on a 150 × 2 mm i.d., 4 µm, 80 Å, Synergi Hydro-RP column connected with a 4 × 2 mm i.d. guard column packed with the same material (Phenomenex, Lane Cove, NSW, Australia). The column temperature was maintained at 20 °C during the HPLC run. A binary gradient with mobile phases consisting of 0.5% formic acid in water (solvent A) and 0.5% formic acid in acetonitrile (solvent B) was used. The elution conditions were as follows: a flow rate of 400 µL/min, a linear gradient of solvent B held at 0% for 5 min, from 0 to 5% in 5 min, from 5% to 20% in 5 min, from 20% to 100% in 2 min and from 100% to 0% for 13 min. A 5 min re-equilibration time at 0% of solvent B was programmed between runs. The effluent from the column was monitored by DAD acquiring absorbance at wavelengths of 254, 280 and 320 nm, followed by introduction to a mass spectrometer through an electrospray (ESI) interface.

For LCMS analysis, a 4000 QTRAP mass spectrometer equipped with a Turbo ion source (Sciex, Mt Waverly, Victoria, Australia) was connected to the outlet of DAD with a PEEK tubing (0.13 mm i.d.). Data acquisition and processing were performed using Analyst software 1.6.2 (Sciex, Mt Waverley, VIC, Australia). ESI negative ion mass spectra were recorded in scan mode ranging from *m*/*z* 50 to *m*/*z* 1000 with a scan time of 1 s and step size of 0.1 Da. Nitrogen was used for curtain, nebulizer and heated turbo (500 °C) gases set at 20 psi, 50 psi and 50 psi, respectively. The electrospray and declustering potentials were set at −4200 V and −50 V, respectively.

The LC-HRMS analysis was performed on an Agilent HPLC 1200 coupled to a Bruker microTOF-Q II (Bruker Singapore, The Helios, Singapore). Mass calibration was performed on each file using Bruker Daltonic’s DataAnalysis v4.1 “Enhanced Quadratic” calibration method (Bruker Singapore, The Helios, Singapore). All other data analysis including chemical formula predictions was done using Bruker Daltonic’s DataAnalysis 4.3. Data were acquired using ESI negative ionisation in scan mode from *m*/*z* 50 to 1650 with a scan speed of 2 spectra/s. Nitrogen was used for the nebulizer and dry gas (200 °C) which were set at 2 bar and 7 L/min, respectively. The capillary voltage was 3500 V and endplate offset was −500 V. DAD acquisition range was 200–600 nm.

#### 4.3.6. Gas Chromatography Coupled to Sulfur Chemiluminescence Detection

All H_2_S measurements in the Verdelho and Shiraz base wines, as well as the treated samples, were determined using an Agilent 355 sulfur chemiluminescence detector (SCD) coupled to an Agilent 6890A gas chromatograph (Forest Hill, VIC, Australia). Experimental and analytical parameters, as well as the analysis method were as described by Siebert et al. [28] without modification.

### 4.4. Sample Preparation and Analysis

#### Preparation of Verdelho, Shiraz, and Model Wine Samples Spiked with Cu^2+^ + SO_2_

Wine samples and all stock solutions were prepared in a low oxygen atmosphere by making use of an anaerobic bag (Aldrich^®^ AtmosBag, Sigma-Aldrich, Castle Hill, NSW, Australia) fitted with a PreSens Pst6 oxygen sensor (Presens, Regensburg, Germany). The bag was flushed down to <1 ppb oxygen with N_2 (g)_ at the start of the sample preparation and a slight positive pressure was maintained to prevent oxygen ingress into the bag during experimental setup. The oxygen concentration in the anaerobic bag was continuously monitored and maintained at <15 ppb. The Verdelho, Shiraz, and model (12% EtOH, 10 g/L tartaric acid, pH = 3.4) base wines were placed inside the anaerobic bag and sub-sampled by placing 10 mL of wine into a 20 mL crimp top amber vial (Chromacol, Thermo Fisher Scientific Inc., Scoresby, VIC, Australia). The Verdelho and Shiraz wines were each divided into four treatments repeated in triplicate (*n* = 12): (1) Cu^2+^ treatment (1 mg/L for Verdelho, Shiraz and model wines); (2) SO_2_ treatment (Verdelho: free 87.8 mg/L, total 100.0 mg/L; Shiraz: free 40.8 mg/L, total 60 mg/L; model wine: free 99.67 mg/L, total 100 mg/L); (3) “Cu^2+^ + SO_2_” treatment, where the above mentioned levels of Cu^2+^ and SO_2_ were combined into one treatment for Verdelho, Shiraz, and model wines; and (4) the control Verdelho, Shiraz, and model wine samples were not treated with either Cu^2+^ or SO_2_ (Table 3).

Stock solutions of Cu^2+^, and SO_2_ were prepared in such a manner that the high level of each metal was added by spiking 50 µL of the appropriate stock solution to the 10 mL wine sample using a pipette, avoiding significant dilution effects. After the samples were prepared with the four treatments, they were sealed with 20 mm magnetic crimp caps with 8 mm center and blue PTFE/silicon septa (118 mm) (Grace Davison Discovery Sciences, Rowville, VIC, Australia) and stored at room temperature (20 °C) in 19 L post mix Cornelius kegs (Ambar technology, Alexandria, NSW, Australia). The lid of each keg was fitted with a PreSens Pst6 oxygen sensor (Presens, Regensburg, Germany). The kegs were flushed with N_2 (g)_ until they reached <1 ppb oxygen and maintained at a slight positive pressure of 1 psi N_2 (g)_ to prevent any oxygen ingress into the keg storage systems. The kegs were continuously monitored and not allowed to exceed an oxygen measurement of 25 ppb, and flushed with N_2 (g)_ to <1 ppb if increased oxygen concentrations were measured.

Verdelho, Shiraz, and model wine samples were analyzed for their VSC concentrations immediately after spiking with metals (Day 0), and then following 1 month (Month 1), 3 months (Month 3), 6 months (Month 6) and 12 months (Month 12) of storage under low oxygen conditions.

### 4.5. Statistical Analyses

All significance tests (Student’s *t*-test, ANOVAs and Tukey analyses) were conducted using GraphPad Prism statistics software (v6.04 GraphPad Software Inc., La Jolla, CA, USA). Multi-way analysis of variance (ANOVA) and Dunnett multiple comparison post-hoc tests were used to determine which treatments were associated with significant effects on the formation of H_2_S over the course of the 12-month experiment, and statistical significance was assigned if *p* < 0.05. All values are represented as mean ± standard deviation (SD).

## Figures and Tables

**Table 1:** Effects of Cu^2+^, SO_2_, and “Cu^2+^ + SO_2_” treatments on H_2_S formation in Verdelho and Shiraz wine samples.

Verdelho Wine Samples											
H_2_S (μg/L)	DAY 0		MONTH 1		MONTH 3		MONTH 6		MONTH 12		Overall Significance
	AVG ^1^	SD ^2^	AVG	SD	AVG	SD	AVG	SD	AVG	SD	
**Control**	1.122	0.040	1.270	0.026	1.066	0.136	2.058	0.012	0.879	0.028	
**Cu^2+^**	0.981	0.062	1.523	0.062	2.276	0.428	10.53	3.776	0.923	0.395	ns
**SO_2_**	1.538	0.042	3.764	2.868	2.139	0.235	5.991	5.312	2.318	0.222	ns
**Cu^2+^ + SO_2_**	1.338	0.137	16.59	1.527	26.64	0.683	50.62	7.685	36.01	18.09	**** (+) ^3^
**Shiraz Wine Samples**											
**H_2_S (μg/L)**	**DAY 0**		**MONTH 1**		**MONTH 3**		**MONTH 6**		**MONTH 12**		**Overall Significance**
	**AVG**	**SD**	**AVG**	**SD**	**AVG**	**SD**	**AVG**	**SD**	**AVG**	**SD**	
**Control**	2.271	0.058	1.672	0.166	2.474	0.241	2.604	0.033	1.515	0.195	
**Cu^2+^**	2.063	0.066	4.202	0.354	14.71	1.701	28.50	1.048	12.41	11.00	**** (+) ^4^
**SO_2_**	2.254	0.053	2.900	0.615	3.162	0.032	2.676	0.244	2.095	0.576	ns
**Cu^2+^ + SO_2_**	2.096	0.101	18.31	0.886	4.594	0.977	25.77	2.364	27.49	10.75	**** (+) ^4^

^1^ AVG: Average values for three replicates; ^2^ SD: Standard deviation values for three replicates; ^3^ *p*-value is the result of significance for the effect, “****” indicates *p*-value ≤ 0.001; ^4^ Significant effect associated with “Cu^2+^” treatment.

**Table 2:** Mass spectral data and retention times of adducts of 4-methyl benzoquinone with H_2_S and SO_2_.

H_2_S-4MC and SO_2_-4MC Adducts ^1^	Measured HRMS ^2^	Predicted HRMS ^3^	Prediction Accuracy (err, mDa) ^4^	Published HRMS ^5^	Peak Ratio ^6^
**H_2_S-4MC adducts: 1mM 4MBQ + 4mM H_2_S**					
4MC dimer + SH; C_14_H_13_O_4_S	277.0543	277.054	−0.3	277.0524	5
4MC dimer + SH; C_14_H_13_O_4_S	277.0542	277.054	−0.2	277.0524	3
4MC dimer + 2SH; C_14_H_13_O_4_S_2_	309.0259	309.0261	0.1	309.0240	1
**SO_2_-4MC adducts: 1 mM 4MBQ + 4 mM SO_2_**					
4MC + SO_3_; C_7_H_7_O_5_S	203.0024	203.0020	−0.4	203.0019	2
4MC + SO_3_; C_7_H_7_O_5_S	203.0022	203.0020	−0.2	203.0019	1
4MC dimer + SO_3_; C_14_H_13_O_7_S	325.0389	325.0387	−0.1	n/a	3
4MC dimer + SO_3_; C_14_H_13_O_7_S	325.0381	325.0387	0.7	n/a	1
**H_2_S-4MC and SO_2_-4MC adducts: 1 mM 4MBQ + 2 mM H_2_S + 2 mM SO_2_**					
4MC dimer + SH; C_14_H_13_O_4_S	277.0542	277.054	−0.2	277.0524	4
4-MC + SO_3_; C_7_H_7_O_5_S	203.0022	203.0020	−0.2	203.0019	1
4MC dimer + SO_3_; C_14_H_13_O_7_S	325.0389	325.0387	−0.1	n/a	3
4MC dimer + SO_3_; C_14_H_13_O_7_S	325.0381	325.0387	0.7	n/a	1
**H_2_S-4MC and SO_2_-4MC adducts: wine relevant concentrations**					
4MC + SO_3_; C_7_H_7_O_5_S	203.0022	203.0020	−0.2	203.0019	1
4MC dimer + SO_3_; C_14_H_13_O_7_S	325.0389	325.0387	−0.1	n/a	5
4MC dimer + SO_3_; C_14_H_13_O_7_S	325.0390	325.0387	−0.2	n/a	1
**H_2_S-4MC and SO_2_-4MC adducts: wine relevant concentrations + metal ions**					
4MC + SO_3_; C_7_H_7_O_5_S	203.0024	203.0020	−0.4	203.0019	1
4MC dimer + SO_3_; C_14_H_13_O_7_S	325.0389	325.0387	−0.1	n/a	2
4MC dimer + SO_3_; C_14_H_13_O_7_S	325.0381	325.0387	0.7	n/a	1

^1^ All 4MC-adducts were tentatively identified using synthesized adducts for comparison of mass spectral data and by comparison with published results [21]; ^2^ Measured HRMS results; ^3^ Predicted molecular mass; ^4^ Accuracy of prediction of molecular formula, error value (mDa) should be <5 mDa for accurate prediction of molecular formula; ^5^ As published by Nikolantonaki et al. [21]; ^6^ Peak ratio measured from the main reaction product peaks of 4MC-adducts.

**Table 3:** Concentrations of Cu^2+^ and SO_2_ in the base wines, spiked samples and average metal and SO_2_ concentrations for Australian wines.

	Base Concentration		Spiked Concentration			Averages	Concentration Range	Legal Limits	
	Verdelho	Shiraz	Verdelho	Shiraz	Model Wine			White Wine ^2^	Red Wine ^2^
Cu^2+^ (mg/L)	0.026	0.015	1.01	0.99	1.00	0.15 ^1^	0.00–1.89 ^1^	1	1
Total/free SO_2_ (mg/L)	<4.00/<4.00	<4.00/<4.00	98.3/87.7	60.7/40.8	100/99.67	74.0 ^3^	10.0 > 350 ^3^	200	150

^1^ Median and concentration ranges for Cu, adapted from Martin et al. [34]; ^2^ Requirements for the export of Australian wine to the European Union [35]; ^3^ Average concentration and concentration range for SO_2_ as measured in >4000 American wines [36].

## Supplementary Materials

The following are available online at http://www.mdpi.com/1420-3049/21/9/1214/s1, Figure S1: Residual H_2_S concentrations measured in samples that replicated wine-like conditions, with 4MBQ at 0.45 mM, H_2_S at 0.39 µM, and SO_2_ 0.23 mM.; as well as the 4MBQ (0.45 mM), H_2_S (0.39 µM), and SO_2_ (0.23 mM) with added Cu^2+^ (0.5 mg/L) and Fe^3+^ (4.0 mg/L), Figure S2: Dissolved oxygen consumption measured from Day 0 to Month 12 for: (**a**) Verdelho samples; and (**b**) Shiraz samples.

## Abbreviations

The following abbreviations are used in this manuscript:

4MBQ	4-methylbenzoquinone
4MC	4-Methylcatechol
ANOVA	Analysis of variance
AVG	Average
AWRI	Australian Wine Research Institute
DO	Dissolved oxygen
GSH	Glutathione
H_2_O_2_	Hydrogen peroxide
H_2_S	Hydrogen sulfide
HPLC	High pressure liquid chromatography
LCMS	Liquid chromatography-mass spectrometry
LC-HRMS	Liquid chromatography-high resolution mass spectrometry
MeSH	Methanethiol
MeOH	Methanol
OT	Odor threshold
PMS	Potassium hydrogen tartrate
SO_2_	Sulfur dioxide
SD	Standard deviation
THF	Tetrahydrofuran
VSCs	Volatile sulfur compounds

## References

[1] Smith M.E., Bekker M.Z., Smith P.A., Wilkes E.N. (2015). Sources of volatile sulfur compounds in wine. Aust. J. Grape Wine Res..

[2] Siebert T.E., Bramley B., Solomon M.R. (2009). Hydrogen sulfide: Aroma detection threshold study in white and red wine. AWRI Tech. Rev..

[3] Villamor R.R., Ross C.F. (2013). Wine matrix compounds affect perception of wine aromas. Annu. Rev. Food Sci. Technol..

[4] Ugliano M., Kwiatkowski M., Vidal S., Capone D., Siebert T., Dieval J.B., Aagaard O., Waters E.J. (2011). Evolution of 3-mercaptohexanol, hydrogen sulfide, and methyl mercaptan during bottle storage of Sauvignon blanc wines. Effect of glutathione, copper, oxygen exposure, and closure-derived oxygen. J. Agric. Food Chem..

[5] Bekker M.Z., Day M.P., Holt H., Wilkes E., Smith P.A. (2016). Effect of oxygen exposure during fermentation on volatile sulfur compounds in Shiraz wine and a comparison of strategies for remediation of reductive character. Aust. J. Grape Wine Res..

[6] Day M.P., Schmidt S.A., Smith P.A., Wilkes E.N. (2015). Use and impact of oxygen during winemaking. Aust. J. Grape Wine Res..

[7] Franco-Luesma E., Ferreira V. (2016). Reductive off-odors in wines: Formation and release of H_2_S and methanethiol during the accelerated anoxic storage of wines. Food Chem..

[8] Franco-Luesma E., Ferreira V. (2016). Formation and release of H_2_S, methanethiol, and dimethylsulfide during the anoxic storage of wines at room temperature. J. Agric. Food Chem..

[9] Nedjma M., Hoffmann N. (1996). Hydrogen sulfide reactivity with thiols in the presence of copper(II) in hydroalcoholic solutions cognac brandies: Formation of symmetrical and unsymmetrical dialkyl trisulfides. J. Agric. Food Chem..

[10] Viviers M.Z., Smith M.E., Wilkes E., Smith P. (2013). Effects of five metals on the evolution of hydrogen sulfide, methanethiol, and dimethyl sulfide during anaerobic storage of Chardonnay and Shiraz wines. J. Agric. Food Chem..

[11] Walker M.D. (1995). The influence of metal-ions on concentrations of flavor-active sulfur-compounds measured in beer using dynamic headspace sampling. J. Sci. Food Agric..

[12] Kreitman G.Y., Danilewicz J.C., Jeffery D.W., Elias R.J. (2016). Reaction mechanisms of metals with hydrogen sulfide and thiols in model wine. Part 1: Copper-catalyzed oxidation. J. Agric. Food Chem..

[13] Clark A.C., Grant-Preece P., Cleghorn N., Scollary G.R. (2015). Copper(II) addition to white wines containing hydrogen sulfide: Residual copper concentration and activity. Aust. J. Grape Wine Res..

[14] Clark A.C., Kontoudakis N., Barril C., Schmidtke L.M., Scollary G.R. (2016). Measurement of labile copper in wine by medium exchange stripping potentiometry utilising screen printed carbon electrodes. Talanta.

[15] Bekker M.Z., Mierczynska-Vasilev A., Smith P.A., Wilkes E.N. (2016). The effects of pH and copper on the formation of volatile sulfur compounds in Chardonnay and Shiraz wines post-bottling. Food Chem..

[16] Jackson R.S. (2008). Wine Science Principles and Applications.

[17] Danilewicz J.C. (2011). Mechanism of autoxidation of polyphenols and participation of sulfite in wine: Key role of iron. Am. J. Enol. Vitic..

[18] Elias R.J., Waterhouse A.L. (2010). Controlling the fenton reaction in wine. J. Agric. Food Chem..

[19] Danilewicz J.C. (2013). Reactions involving iron in mediating catechol oxidation in model wine. Am. J. Enol. Vitic..

[20] Waterhouse A.L., Laurie V.F. (2006). Oxidation of wine phenolics: A critical evaluation and hypotheses. Am. J. Enol. Vitic..

[21] Nikolantonaki M., Waterhouse A.L. (2012). A method to quantify quinone reaction rates with wine relevant nucleophiles: A key to the understanding of oxidative loss of varietal thiols. J. Agric. Food Chem..

[22] Nikolantonaki M., Magiatis P., Waterhouse A.L. (2014). Measuring protection of aromatic wine thiols from oxidation by competitive reactions vs wine preservatives with ortho-quinones. Food Chem..

[23] Rankine B.C. (2004). Making Good Wine.

[24] Ribereau-Gayon P., Glories Y., Maujean A., Dubourdieu D. (2006). Handbok of Enology: The Chemistry of Wine Stabilization and Treatments.

[25] Lopes P., Silva M.A., Pons A., Tominaga T., Lavigne V., Saucier C., Darriet P., Teissedre P.L., Dubourdieu D. (2009). Impact of oxygen dissolved at bottling and transmitted through closures on the composition and sensory properties of a sauvignon blanc wine during bottle storage. J. Agric. Food Chem..

[26] Nikolantonaki M., Jourdes M., Shinoda K., Teissedre P.L., Quideau S., Darriet P. (2012). Identification of adducts between an odoriferous volatile thiol and oxidized grape phenolic compounds: Kinetic study of adduct formation under chemical and enzymatic oxidation conditions. J. Agric. Food Chem..

[27] Ugliano M. (2013). Oxygen contribution to wine aroma evolution during bottle aging. J. Agric. Food Chem..

[28] Siebert T.E., Solomon M.R., Pollnitz A.P., Jeffery D.W. (2010). Selective determination of volatile sulfur compounds in wine by gas chromatography with sulfur chemiluminescence detection. J. Agric. Food Chem..

[29] Danilewicz J.C. (2007). Interaction of sulfur dioxide, polyphenols, and oxygen in a wine-model system: Central role of iron and copper. Am. J. Enol. Vitic..

[30] Danilewicz J.C., Seccombe J.T., Whelan J. (2008). Mechanism of interaction of polyphenols, oxygen, and sulfur dioxide in model wine and wine. Am. J. Enol. Vitic..

[31] Rauhut D., Kurbel H., Dittrich H.H. (1993). Sulfur compounds and their influence on wine quality. Wein Wiss..

[32] Franco-Luesma E., Ferreira V. (2014). Quantitative analysis of free and bonded forms of volatile sulfur compouds in wine. Basic methodologies and evidences showing the existence of reversible cation-complexed forms. J. Chromatogr. A.

[33] Thiel G., Geisler G., Blechschmidt I., Danzer K. (2004). Determination of trace elements in wines and classification according to their provenance. Anal. Bioanal. Chem..

[34] Martin A.E., Watling R.J., Lee G.S. (2012). The multi-element determination and regional discrimination of Australian wines. Food Chem..

[35] (2016). Analytical Requirements for the Export of Australian Wine. http://www.awri.com.au/industry_support/regulatory_assistance/export_requirements/.

[36] Peterson G.F., Kirrane M., Hill N., Agapito A. (2000). A comprehensive survey of the total sulfur dioxide concentration of American wines. Am. J. Enol. Vitic..

[37] Jongberg S., Gislason N.E., Lund M.N., Skibsted L.H., Waterhouse A.L. (2011). Thiol-quinone adduct formation in myofibrillar proteins detected by lc-ms. J. Agric. Food Chem..

[38] Fisher U., Noble A.C. (1994). The effect of ethanol, catechin concetration, and pH on sourness and bitterness of wine. Am. J. Enol. Vitic..

[39] Komes D., Ulrich D., Kovacevic G., Lovric T. (2007). Study of phenolic and volatile composition of white wine during fermentaion and a short time of storage. Vitis.

[40] Mercurio M.D., Dambergs R.G., Herderich M.J., Smith P.A. (2007). High throughput analysis of red wine and grape phenolics-adaptation and validation of methyl cellulose precipitable tannin assay and modified Somers color assay to a rapid 96 well plate format. J. Agric. Food Chem..

