# Supplementary material for: Formation of Hydrogen Sulfide in Wine: Interactions between Copper and Sulfur Dioxide
Source: Molecules. 2016 Sep 10;21(9):1214. doi: 10.3390/molecules21091214 (PMC6274298; doi:10.3390/molecules21091214)
Supplement: Supplementary file 1 [file molecules-21-01214-s001.pdf]

Supplementary Materials: Formation of Hydrogen Sulfide in Wine: Interactions between Copper and Sulfur Dioxide

Marlize Z. Bekker, Mark E. Smith, Paul A. Smith and Eric Wilkes

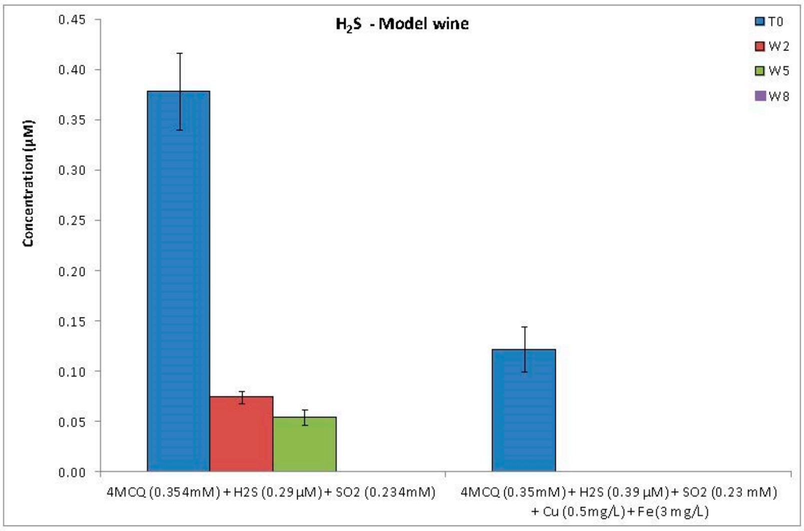

Figure S1. Residual H_2S concentrations measured in samples that replicated wine-like conditions, with 4MBQ at 0.45 mM, H_2S at 0.39 µM, and SO_2 0.23 mM.; as well as the 4MBQ (0.45 mM), H_2S (0.39 µM), and SO_2 (0.23 mM) with added Cu^{2+} (0.5 mg/L) and Fe^{3+} (4.0 mg/L).

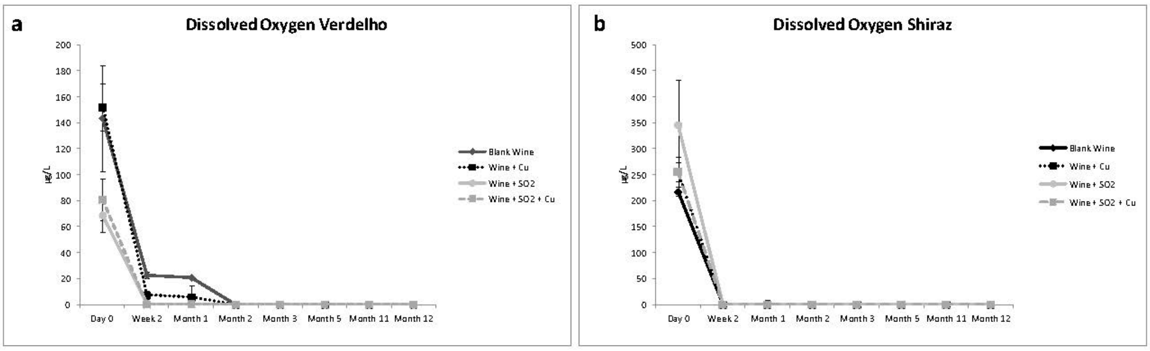

Figure S2. Dissolved oxygen consumption measured from day 0 to month 12 for (**a**) Verdelho samples and (**b**) Shiraz samples.
